# The Special Collections Handbook. 2nd ed

**DOI:** 10.5195/jmla.2018.415

**Published:** 2018-04-01

**Authors:** Paul M. Blobaum

**Affiliations:** Archives and Special Collections, University Library, Governors State University, University Park, IL

It is difficult to believe that the first edition of this book in 2011 was the first comprehensive single volume manual to appear in special collections librarianship. Professionals and others working with special collections and archival materials typically have had to undertake time-consuming research to find needed information, consulting the web, journal articles, and monographs that often are filled with theory or irrelevant details. The *Special Collections Handbook* by Alison Cullingford potentially puts everything that a librarian would need in a concise overview and additional resources on the web at one’s fingertips, but for international audiences outside of the United Kingdom, additional research must be done to find comparable resources that are specific to the country of origin. Cullingford pioneered the role of special collections librarian in 2000 at the University of Bradford, in the city of Bradford, West Yorkshire, England. This book is based on her more than fifteen years of practice of building a special collections program from scratch.

In Cullingford’s world, the professional is not merely concerned with preserving special collections materials in the library. Those materials must be showcased and promoted, and new constituencies and supporters must be identified. Published in the United Kingdom and written from a British perspective, Cullingford’s book keeps a busy working professional in mind, especially those who have multiple assignments in a library; have hidden, unprocessed, or donated collections accrued over hundreds of years; and are in need of a brief overview and pep talk on what needs to be done. Consideration is also given to the international audience, providing references to both European and American resources.

Cullingford’s intention is to give readers essential information, illustrated with examples and case studies, to consider and render conclusions. Bibliographies and web sources are provided at the end of each chapter for more reading. The author is aware that most readers will find themselves either improving or building a special collections program, writing policies and procedures, or rallying supporters, and thus, they are looking for sound, practice-based advice.

The second edition follows the original framework of discussing collection care, emergency planning, understanding of special collections materials, collection development and description, marketing, access and legal considerations, and fund-raising. These chapters have been revised and joined by two new chapters on digitization and organizational issues for storing collections and people to manage them.

The author clearly takes delight in sharing with readers practical advice and essential points to consider in nearly every conceivable aspect of rare books librarianship, providing a framework for early career professionals as well as volunteers and collectors of materials. A companion blog solves the problem of link rot: web resources and new additions are kept current, and additional materials are posted. Seasoned professionals will appreciate this book and companion blog as a pep talk, review, and ready reference.

International readers will need to look beyond the book’s parochialisms to identify locally relevant resources, laws and regulations, and standards. Some work must be done to translate unfamiliar acronyms and metric measures, such as temperature readings. A detailed index helps solve some mysteries, but a Google search may need to be performed. Readers may be disappointed with the brevity of some of the material and will have to overlook the book’s main focus on the needs of readers in the United Kingdom. Facet and other publishers are trying to reach US audiences, but often the books are not practical for librarians with limited budgets. Facet Publishing and authors would go far to win over the North American audience by publishing US- and Canada-specific web resources for each chapter on a supplemental blog or wiki, or by considering an edition revised specifically for these markets.

Cullingford welcomes recommendations of websites for her companion blog, but readers should not have to rely on reader input to make the book practical for North American readers. The book falls short of its potential for the international audience, and its purchase is discretionary at its US $85 price. This book should be considered a companion textbook for rare books, special collections, preservation, and archives management courses in library schools.

